# Dual-Energy Computed Tomography Proton-Dose Calculation with Scripting and Modified Hounsfield Units

**DOI:** 10.14338/IJPT-20-00075.1

**Published:** 2021-06-25

**Authors:** Anthony Kassaee, Chingyun Cheng, Lingshu Yin, Wei Zou, Taoran Li, Alexander Lin, Samuel Swisher-McClure, John N. Lukens, Robert A. Lustig, Shannon O'Reilly, Lei Dong, Roni Hytonen MS, Boon-Keng Kevin Teo

**Affiliations:** 1 Department of Radiation Oncology, University of Pennsylvania, Philadelphia, PA, USA; 2 Department of Radiation Oncology at Rutgers Cancer Institute of New Jersey, New Brunswick, NJ, USA; 3 Varian Medical Systems, Palo Alto, CA, USA

**Keywords:** dual-energy CT, proton therapy, stopping-power ratios

## Abstract

**Purpose:**

To describe an implementation of dual-energy computed tomography (DECT) for calculation of proton stopping-power ratios (SPRs) in a commercial treatment-planning system. The process for validation and the workflow for safe deployment of DECT is described, using single-energy computed tomography (SECT) as a safety check for DECT dose calculation.

**Materials and Methods:**

The DECT images were acquired at 80 kVp and 140 kVp and were processed with computed tomography scanner software to derive the electron density and effective atomic number images. Reference SPRs of tissue-equivalent plugs from Gammex (Middleton, Wisconsin) and CIRS (Computerized Imaging Reference Systems, Norfolk, Virginia) electron density phantoms were used for validation and comparison of SECT versus DECT calculated through the Eclipse treatment planning system (Varian Medical Systems, Palo Alto, California) application programming interface scripting tool. An in-house software was also used to create DECT SPR computed tomography images for comparison with the script output. In the workflow, using the Eclipse system application programming interface script, clinical plans were optimized with the SECT image set and then forward-calculated with the DECT SPR for the final dose distribution. In a second workflow, the plans were optimized using DECT SPR with reduced range-uncertainty margins.

**Results:**

For the Gammex phantom, the root mean square error in SPR was 1.08% for DECT versus 2.29% for SECT for 10 tissue-surrogates, excluding the lung. For the CIRS Phantom, the corresponding results were 0.74% and 2.27%. When evaluating the head and neck plan, DECT optimization with 2% range-uncertainty margins achieved a small reduction in organ-at-risk doses compared with that of SECT plans with 3.5% range-uncertainty margins. For the liver case, DECT was used to identify and correct the lipiodol SPR in the SECT plan.

**Conclusion:**

It is feasible to use DECT for proton-dose calculation in a commercial treatment planning system in a safe manner. The range margins can be reduced to 2% in some sites, including the head and neck.

## Introduction

Proton therapy provides significant advantages in dose distribution because of the sharp dose falloff near the end of the proton range. Dose painting with pencil beam-scanning techniques, combined with the inherent Bragg peak from heavy-charged particles, allows for high dose conformity and sparing of organs at risk (OARs). Precise control of the location of individual Bragg peaks requires accurate material composition information within the beam path. The human body is composed of an array of tissues that have varying atomic number and mass density. Current proton-treatment planning systems use single-energy computed tomography (SECT) Hounsfield units (HUs) to estimate proton stopping-power ratios (SPRs) by applying a calibration curve. However, HUs from x-rays are a measure of a material's linear attenuation and do not have a one-to-one correspondence with SPRs, resulting in errors in the SPR calculation.

One of the most common HU-to-SPR calibration procedures is the stoichiometric method introduced by Schneider et al [1]. In that method, the computed tomography (CT) scanner is calibrated to a specific x-ray spectrum, and the CT-to-SPR calibration curve is created by applying the calibrated model to a set of human reference tissues. However, there are uncertainties that arise from CT imaging that translate to dose-calculation uncertainties [2] because of the estimation error in human-tissue SPRs. To account for SPR uncertainties, a range margin, typically between 3% and 3.5% of the proton range, is applied to the target volume in the beam direction, resulting in additional radiation to healthy tissues.

Dual-energy CT (DECT) has been shown to increase the accuracy of calculated SPRs from CT scanners [3]. The DECT acquires 2 CT images with different energies to exploit the differential response of materials to varying energies of the x-ray spectra through the photoelectric effect, which has high atomic number and energy dependence. Dual-energy computed tomography has been used to differentiate materials since the 1970s when Hounsfield [4] observed that 2 images taken at different energies could show the differences between iodine (*Z* = 53) and calcium (*Z* = 20) concentrations. Moreover, DECT can provide additional material-composition information, such as the relative electron density to that of water (ρ*_e_*) and the effective atomic number (*Z*_eff_). There are various DECT methods for extracting the electron density and *Z*_eff_ from the DECT image to derive proton SPR. A summary comparing the different methods can be found in Bar et al [5, 6]. With better information about material compositions, proton SPR calculations are enhanced, allowing for greater accuracy in dose calculations and the potential of reducing the range-uncertainty margin compared with that of SECT.

Studies have suggested improving the stoichiometric calibration method using DECT scans. Many studies note that there is an improvement when using DECT scans over SECT for more-accurate dose distributions. Yang et al [3] noted in their study that their DECT method using electron-density ratios and the *Z*_eff_ was less sensitive to variations in density and elemental compositions than the previously existing clinical methods using the stoichiometric calibration [2]. Planning studies [7, 8] have demonstrated the potential dosimetric superiority of DECT compared with SECT. The first clinical application of DECT in proton planning [9] used pseudomonoenergetic CT images in place of SECT for the HU-to-SPR conversion, rather than using the derived-material information ρ*_e_* and *Z*_eff_ afforded by DECT to calculate the SPR [3]. Another approach in clinical implementation of DECT used the calculated SPRs to modify the SECT HU-to-SPR calibration curve [10]. Changes to that curve allowed for more-accurate SPR results and allowed the clinic to follow the same workflow as previously used with SECT. In this article, we demonstrate a method for DECT implementation that uses the same SECT HU-to-SPR curve but with more-accurate HU values calculated with a script in the planning system. This allows the planning system to follow the same clinical workflow previously used but with improved proton-range predictions and dose calculations from DECT.

## Materials and Methods

### DECT Scanner and Planning System Script

The DECT images in this study were acquired on a Definition Edge CT scanner (Siemens Healthineers, Malvern, Pennsylvania) with the sequential scan mode at 80 kVp, followed by 140 kVp. Iterative image reconstruction was used to reduce the image noise, and a slice thickness of 2 mm was used for head and neck scans [11]. A mixed image was derived from a linear combination of the 80-kVp (30%) and the 140-kVp (70%) images to mimic a 120-kVp SECT image for comparison with DECT planning. The ρ*_e_* and *Z*_eff_ images were generated from the DECT image with the Siemens syngo.via image processing software. The mixed image, as well as the ρ*_e_* and *Z*_eff_ images, were imported into the Eclipse treatment planning system V16 (Varian Medical Systems, Palo Alto, California) for dose calculation.

**Figure 1.**
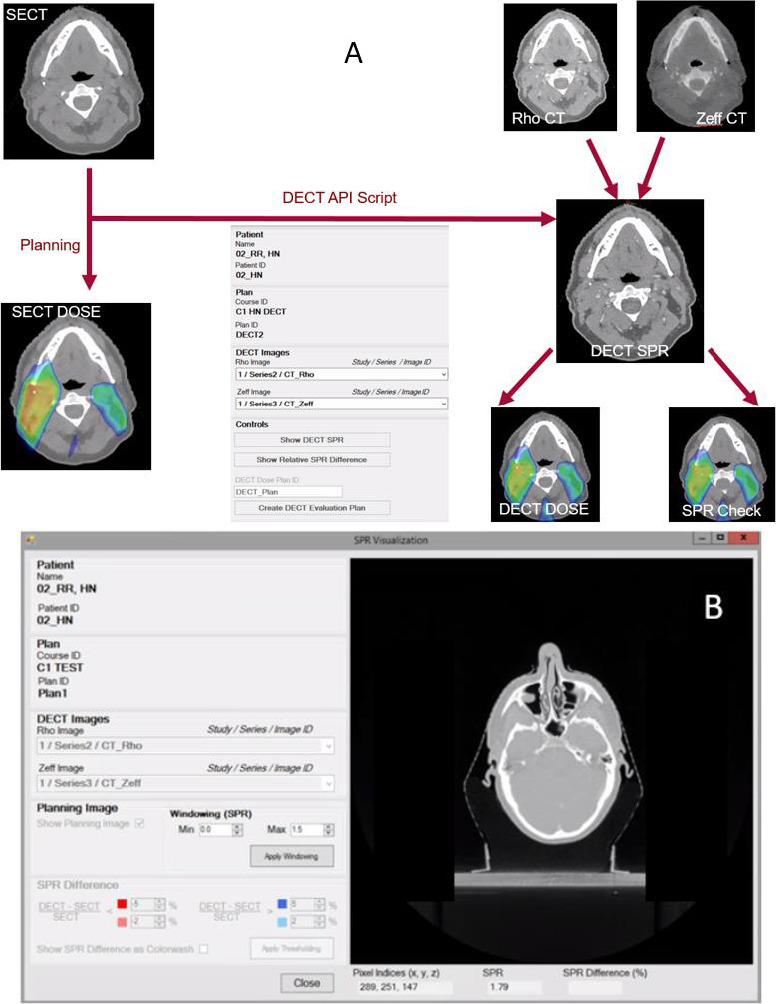
(A) shows the workflow for calculating the dual-energy computed tomography (DECT)-based dose distribution with the Varian DECT Eclipse scripting application programming interface (ESAPI) script. Plan optimization is performed using the single-energy computed tomography (SECT) image. (B) A sample readout tool of the DECT stopping-power ratio (SPR) using the DECT ESAPI script.

As a safety measure, the SECT image was used first for optimization to create a traditional SECT-based plan and dose distribution, as seen on the left in the flowchart in **Figure 1A**. The DECT script was then run, using the Eclipse scripting application programming interface (ESAPI), which generates a DECT-calculated SPR image internally using the ρ*_e_* and *Z*_eff_ images. For comparison purposes with the SECT image, the SPR image was inverted back into HUs using the SECT HU-SPR lookup table. As part of the script, DECT-computed SPR can be inspected at the voxel level as shown in **Figure 1B**. This feature was used for phantom DECT SPR validation, as described in the next section. In the next step of the workflow, the SECT plan, all contours, and the HU overrides were copied internally from the SECT CT onto the DECT SPR CT to generate a forward-calculated DECT dose for comparison with the SECT dose. These override structures are common to both SECT and DECT images and are typically CT-imaging artifacts arising from presence of dental implants or metals and treatment accessories, such as couch structures, which have known water-equivalent thicknesses and, therefore, assigned HUs.

In the workflow described, plan optimization was performed on the SECT image, whereas the DECT SPR was used only to generate the final dose distribution. In a second workflow, the DECT SPR was generated using MATLAB software (The MathWorks, Natick, Massachusetts) and was imported into the Eclipse software for use in plan optimization, whereas the SECT image was used for dose check. The MATLAB script was compared with the ESAPI script for validation of the code.

### Validation of DECT-Calculated SPRs

In this study, the 467 Phantom (Gammex Inc, Middleton, Wisconsin) and the Computerized Imaging Reference Systems (CIRS) Phantom model 062M (CIRS, Norfolk, Virginia) were used to validate the accuracy of DECT-calculated SPRs [12]. Both Phantoms were placed back to back during the CT scan to achieve a total thickness of > 10 cm in the axial direction to ensure reliable DECT-image reconstruction. The GAMMEX and CIRS Phantoms were used for the validation because they each provided a variety of known chemical compositions simulating 8 tissue-surrogate tissue types. The mixed (SECT), ρ*_e_*, and *Z*_eff_ image sets were uploaded to the Eclipse treatment planning system, where the DECT ESAPI script was run. The DECT-SPR readout tool in the ESAPI script (**Figure 1B**) was used to extract the SPR, calculated using the approach of Hünemohr et al [12]:

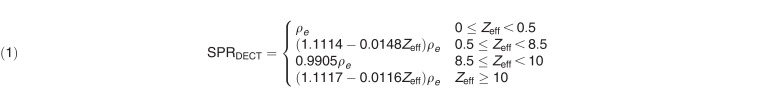



The distinct regions arise from the parameterization of the ionization potential by *Z*_eff_ into different tissue classes. *Z*_eff_ values < 8.5 represent soft tissue, whereas those > 10 represent bony tissue. *Z*_eff_ values from the Siemens software are assigned to 0 in very low density tissues (HU < −500), such as the lung (*Z*_eff_ ≈ 7.5), where the calculation is less reliable. A special condition was added in the SPR calculation for *Z*_eff_ between 0 and 0.5 in those regions so that the calculated SPRs were similar to the SECT values. The SECT SPR-calibration method used for comparison was based on the stoichiometric technique [1, 13].

Reference DECT SPR values were hand calculated using vendor-provided batch-specific elemental-composition information of the Gammex and CIRS Phantoms. The expressions used to calculate ρ*_e_* and *Z*_eff_ are presented in **Equations 2** and **3**, respectively. The SPR is then calculated using the Bloch formula in **Equation 4** using a proton energy of 115 MeV and mean ionization potential for liquid water (*I*_water_) of 75 eV.

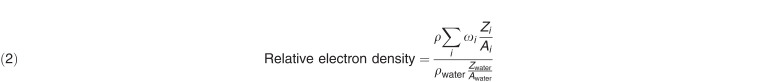


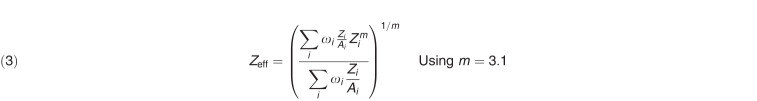


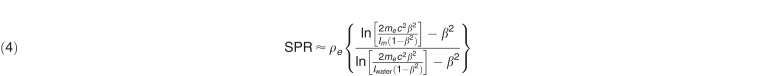



SPR residuals between CT scanner–predicted and reference SPRs were computed. The root-mean-square error (RMSE) was calculated and compared for both DECT and SECT. In addition, the SPRs of the CIRS Phantom tissue-surrogate plugs were measured by irradiation with monoenergetic proton beams for comparison between DECT and SECT predictions. The water-equivalent thicknesses of the plugs were determined from the exit-intensity profiles of the proton beams on the Gafchromic EBT3 film (Ashland, Bridgewater, New Jersey) through the plugs and water reference [14].

The CIRS Phantom was set up with 3 different variations to account for variability in DECT calculations between different sizes and positions. In setup 1, the smaller-circle CIRS Phantom was used to represent a typical patient needing head therapy. In setup 2, the plugs stayed in the same location as those in setup 1, but the entire CIRS Phantom was used to represent a larger patient body part, such as the pelvic region. These 2 setups simulated the effects of beam hardening on the DECT calculation. In setup 3, the entire CIRS Phantom was used, but the plugs were positioned on the outer ring, instead of the inner layer, in the previous 2 setups.

### Clinical Examples of the DECT Script

Images of a postoperative patient with head and neck cancer treated with proton therapy to the primary site and elective neck nodes was used to demonstrate the DECT script and for dosimetric comparisons. The plan used a 3-field arrangement with 2 posterior oblique beams, covering the superior portion of the target, and 1 anterior field beam, covering the lower portion of the target. Multifield robust optimization (RO) was used to cover the 3 clinical target volumes (CTVs) to dose levels of 6300 centigray (cGy), 6000 cGy, and 5400 cGy in 30 fractions. For the workflow using the DECT script, the SECT image was used for optimization with RO parameters of a 3-mm setup and a 3.5% range uncertainty to create the traditional dose distribution (SECT dose). When the script was run, forward calculation of the SECT plan on the internally generated DECT SPR was performed to provide a second dose distribution (DECT dose), which reflected a more-accurate dose map because of the use of DECT SPRs. In a second workflow, the MATLAB-generated DECT SPR was used for plan optimization using RO parameters of 3-mm for setup and 2.0% for range uncertainty. This third dose distribution (DECT_opt dose) was used to evaluate the effect of tighter margins. A second clinical example for liver treatment is presented. The plan was optimized on the SECT image with the single-field optimization technique and consisted of a right lateral and a right posterior oblique field to cover the CTV to 6000 cGy in 20 fractions. The DECT script was then used to evaluate differences in SPR and doses between the DECT and SECT images.

## Results

**Figure 2.**
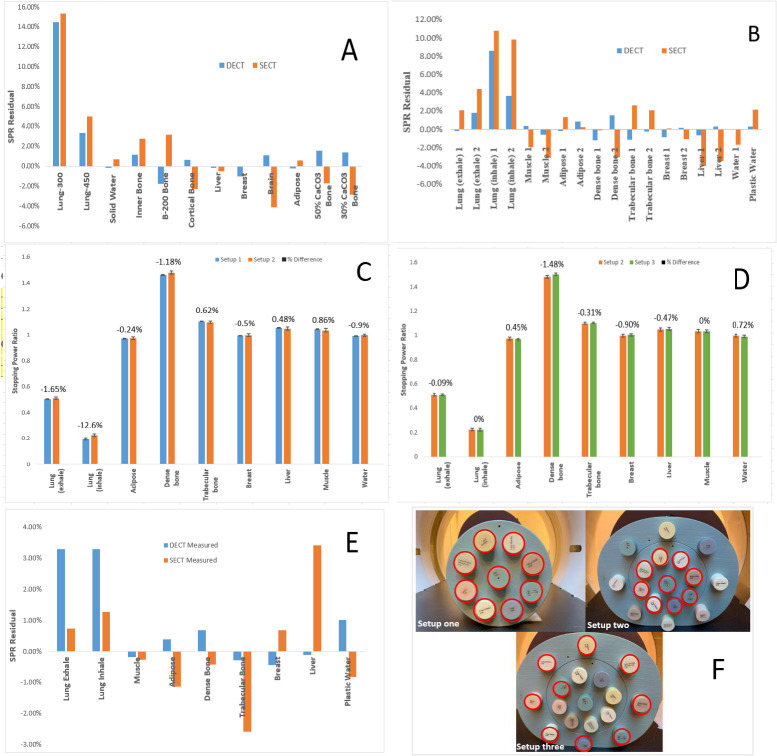
Stopping power ratio (SPR) residuals of dual- and single-energy computed tomography (DECT and SECT)-predicted SPR from reference values for the Gammex Phantom (A) and the Computerized Imaging Reference Systems (CIRS) Phantom (B). Effect of DECT SPR calculation on CIRS Phantom size (setup 1 versus 2) (C) and location of plugs (setup 2 versus 3) (D) within the full phantom. The SPR residuals using SECT and DECT are compared with measured SPRs of the CIRS phantom (E). Images of setups 1, 2, and 3 with the corresponding locations of the plugs circled in red (F).

The MATLAB-calculated SPRs matched the ESAPI-calculated SPRs extracted from the graphic user interface shown in Figure 1 for points sampled on the CIRS Phantom. **Figure 2A** and **2B** shows a comparison of the SPR differences among the DECT- and SECT-predicted SPRs and the reference SPRs based on vendor-provided material-composition data for different tissue surrogates. Compared with SECT, DECT achieved better accuracy to the ground truth for most of the plugs shown in **Figure 2A** and **2B**. Excluding the lung plugs, almost all DECT residuals were under 1%. For the Gammex Phantom, the RMSE was 1.08% for DECT compared with 2.29% for SECT for 10 tissue surrogates, excluding the lungs. For the CIRS Phantom, the corresponding results were 0.74% versus 2.27% for 14 tissue surrogates, excluding the lungs. When compared with the measured SPR results of the CIRS Phantom, the RMSEs were 0.53% for DECT and 1.73% for SECT for 7 tissue surrogates, excluding the lung plugs (**Figure 2E**).

**Figure 2C** compares the DECT SPRs for 2 Phantom sizes, whereas **Figure 2D** shows the variation arising from different plug locations. Most variations were < 1.0%. The measured error bars represent 1 SD inside a region of interest. Excluding the lungs, the RMSE differences in DECT SPRs were because the changing Phantom size was 0.74% and the changing plug location was 0.76%. There was no systematic trend of higher or lower DECT SPR when changing Phantom size or position, but dense bone is most sensitive to variations in Phantom size and plug location.

**Figure 3.**
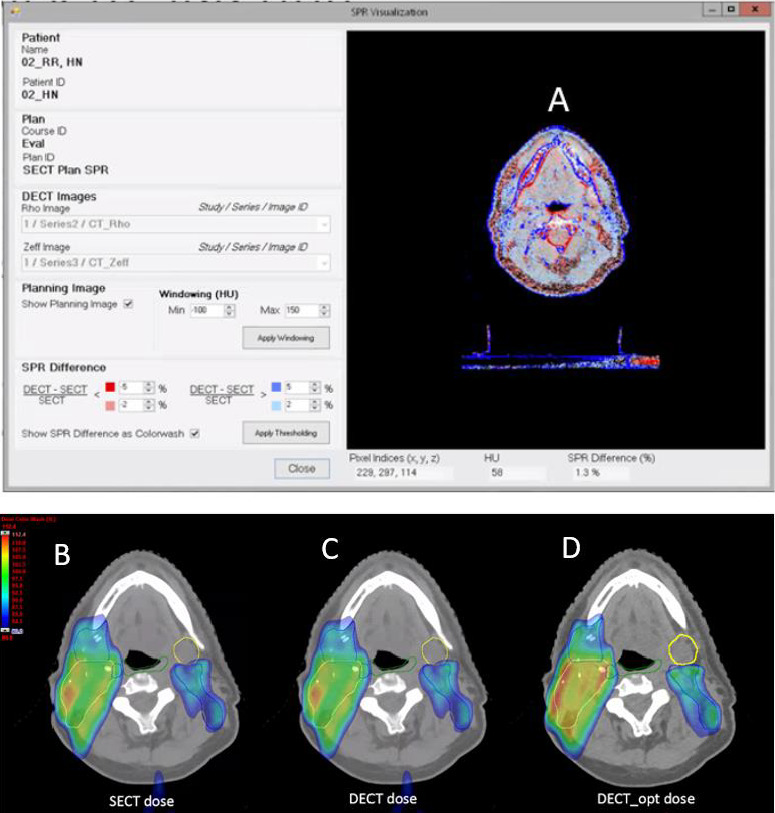
(A) Color map showing stopping power ratio (SPR) differences between dual- and single-energy computed tomography (DECT and SECT) for head and neck treatment. Blue (red) region shows higher (lower) DECT SPR compared with SECT. (B–D) Axial view of head and neck SECT dose (B), the corresponding forward calculated DECT dose (C), and the DECT-optimized dose (D). Yellow and blue target contours are clinical target volume (CTV) 6300 and CTV 5400, respectively. Organ-at-risk (OAR) contours for constrictors (green) and left submandibular (yellow) are shown.

**Figure 4.**
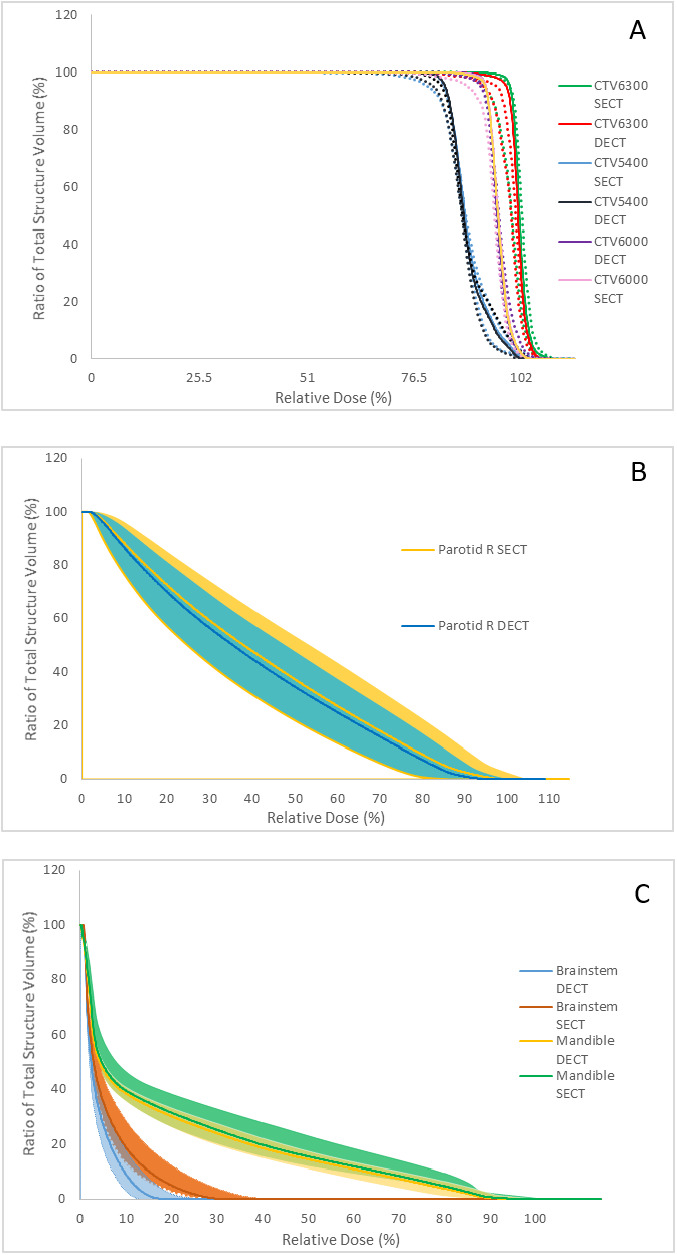
(A) Dose-volume histogram (DVH) of clinical target volume (CTV) 6300, CTV 6000, and CTV 5400 for the single-energy computed tomography (SECT) versus the double-energy computed tomography (DECT) optimized plans. Solid lines are the nominal doses, whereas corresponding dotted lines are the worst-case scenarios. Sample DVH bands for the right parotid (B) and brainstem and mandible (C) are shown for SECT optimization versus DECT optimization. The DVH bands correspond to worst-case scenarios of a 3-mm shift and 3.5% (2%) range uncertainty for the SECT (DECT) plans.

The clinical examples show that the DECT SPR can provide benefits in a variety of ways. As seen in the CIRS Phantom, the muscle SPR was slightly higher in DECT compared with that of SECT. Higher SPR in muscles, but lower SPR in adipose, was also observed in the head and neck DECT compared with that of the SECT in **Figure 3A**. **Figure 3B** and **3C** shows a comparison of the dose distributions between the SECT dose and the script-calculated DECT dose. Because the proton beam path consists primarily of muscle and adipose tissues, the effect of SPR differences between SECT and DECT partially cancel out. The dose-volume histogram (DVH) bands with robustness evaluation are graphed and displayed in Figure 4 comparing SECT optimization with that of DECT optimization. With reduced margins, the uncertainty bands for the DECT plan are smaller than those of the SECT plan. The CTV coverage, even within the DVH bands, remained very similar between the SECT and DECT optimized plans. Major differences can be seen in some of the highlighted OARs in Figure 4. The SECT right parotid uncertainty-dose distribution encompasses all of the uncertainty scenarios for the DECT plan. In that example, the RO was not applied to the OARs, but the uncertainty bands would narrow even further if it was turned on. The expected smaller-dose cloud in the DECT-optimized plan can be seen in the lower-falloff dose in the left submandibular gland when **Figure 3C** and **3D** are compared.

The Table presents a comparison of the SECT dose, DECT dose, and the DECT_opt dose to the OARs. The SECT dose columns represent values based on the SECT plan, whereas the DECT dose columns show values from the SECT plan forward calculated on the DECT image set and reflect more-accurate doses from the SECT plan. The DECT_opt doses represent the DECT-optimized plan with reduced margins. Comparison between the DECT dose and DECT_opt doses show a small improvement in OAR sparing when range-uncertainty margins are reduced.

**Table.** Comparison between absolute single-energy computed tomography (SECT) dose, double-energy computed tomography (DECT) dose, and DECT optimized (DECT_opt) plan dose.

Area under treatment	Mean dose, cGy			Maximum dose, cGy		
	SECT	DECT	DECT_opt	SECT	DECT	DECT_opt
Brainstem	354.2	348.0	241.4	2073.7	2103.3	1294.3
Oral cavity	132.8	131.7	125.4	3106.3	3175.1	2989.3
Constrictors	1643.9	1647.4	1637.1	5853.7	5743.9	5649.1
Left submandibular gland	2232.3	2328.6	2258.5	5155.9	5406.8	5286.3
Larynx	1444.6	1450.9	1424.7	5472.6	5495.2	5516.4
Mandible	1217.8	1204.9	1151.8	6286.0	6186.4	6137.4
Left parotid	1147.4	1142.7	1201.3	5010.1	5046.2	5053.6
Right parotid	2597.0	2570.6	2465.2	6294.0	6230.5	6208.1
Spinal cord	493.6	495.2	491.3	3962.6	3898.6	3474.4

**Figure 5.**
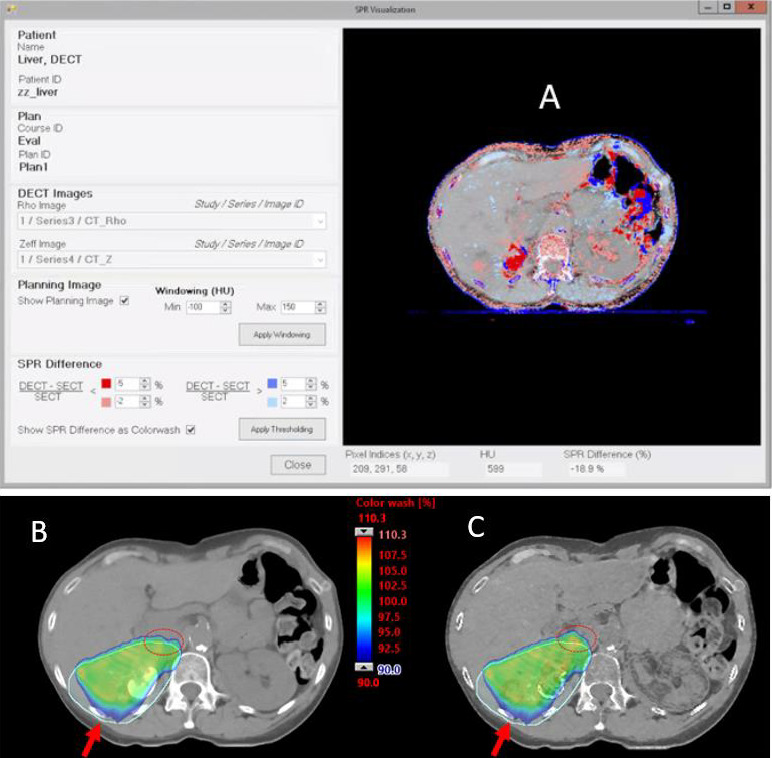
(A) Color map showing stopping power ratio (SPR) differences between dual- and single-energy computed tomography (DECT and SECT) for a patient with liver disease. Blue (red) region shows higher (lower) DECT SPR compared with SECT. (B and C) Comparison of SECT (B) and DECT (C) doses for the posterior oblique fields (red arrow) traversing through a region with dense lipiodol uptake in the liver. Beam overranging (red circle) can be seen in the DECT dose.

For the patient with liver therapy, dense lipiodol uptake is visible in the SECT image. Because lipiodol is an iodinated contrast agent with high HUs, the SPR is overestimated on the SECT plan. It has been shown that DECT can calculate the SPR of contrast materials more accurately than SECT can [15]. The SPR evaluation tool shows that the lipiodol region has approximately 20% lower SPR in the DECT compared with that of the SECT (Figure 5). Because the lipiodol HU was not overridden in the SECT plan, it can be seen in Figure 5, there is a notable shift in the distribution of the right posterior oblique field between the SECT and DECT doses. In this example, the script allows the user to visualize the differences in SPR when comparing SECT and DECT. In some patients, dense objects, such as surgical hardware, can also be seen in the liver. With SECT, it is sometimes hard to distinguish whether the high HU object is lipiodol or other surgical hardware, so it is not definitive that it will always be overridden. This tool helps the user better understand and quantify where the SPR accuracy in the SECT image could be improved, such as by overriding the HUs in lipiodol regions.

## Discussion

Dual-energy CT scanners are now widely available for use in many radiation oncology centers. Although the advantages of DECT over SECT for proton SPR calculation are well known, the translation of the technology to clinical use has been lacking in commercial treatment planning systems. In this work, clinical implementation and workflow using an ESAPI script is demonstrated on the Eclipse treatment planning system. Calculation of the SPR on tissue surrogates using the ESAPI script showed that DECT is more accurate than SECT is. For safe implementation, a workflow using the SECT for plan optimization and the DECT for final dose calculation is presented. The DECT ESAPI script also provided functions for comparison between SECT and DECT SPRs, allowing for better quantification of the differences. Clinical plans further showed the benefit of DECT images, providing a more-accurate dose distribution and allowing reduction of margins in some treatment sites, such as the head and neck. The study showed a reduction of margins from 3.5% to 2% is feasible for head and neck treatment but is likely to also apply to sites not affected by respiratory motion or changes in interfraction organ filling. In cases in which the margins are not reduced, DECT is still beneficial because a more-accurate dose distribution is obtained. An added benefit is that a single-contrast–enhanced DECT scan may be used for dose calculation and obviates the need for a separate noncontrast scan that many proton centers acquire for dose-calculation purposes. Plans robustly optimized for only setup variation can, in practice, slightly compensate for CT number errors. Similarly, robustness in range uncertainty can also help compensate for anatomic uncertainty, such as interfraction setup variability, such as in head and neck scanning. Therefore, the potential effect of target coverage from setup variability and the reproducibility of daily immobilization must be considered before reducing the range-uncertainty margin in robust optimization.

Many patients have foreign objects, such as surgical hardware or implants, which may contain some high-*Z* materials, such as iodine but that, otherwise, have low stopping power. The DECT may be used to help quantify the SPR of these unknown materials, which would otherwise show up as dense objects on SECT, and provide a more-accurate dose distribution for the patient. Further DECT studies will continue to be explored in other treatment areas, such as in patients with foreign implants in the breast. The DECT can more accurately determine the composition of those implants, which may contain small quantities of high-*Z* materials. A reduction of margins will not be feasible in cases of breast cancer because of motion uncertainty, but a more-accurate dose distribution will be achieved using DECT calculations. Lastly, image artifacts from motion, the presence of dense metals, and beam hardening, which affect SECT image quality, also affect DECT SPR accuracy and must be taken into consideration in choosing the appropriate range-uncertainty margins.

In this study, we demonstrated the feasibility and workflow of using DECT for proton-dose calculation in a commercial treatment planning system. The range margins can be reduced to 2% in selected treatment sites, including the brain and head and neck. Using a script for DECT-based SPR calculation allows for better integration with treatment planning systems and comparisons that demonstrate dosimetric differences over the SECT method
